# 

**Published:** 1887-07-30

**Authors:** 


### THE HOSPITAL

**An Institution, Family, and Parochial Journal of Hospitals, Asylums, & all Agencies for the Care of the Sick, Criticism & News.**

### CONTENTS.

**"Sponsors" for Out-Patients** - 291
**Words of Consolation.**—XLIII. The Work of the Holy Ghost - 292
**Hospital Worthies.**—John Radcliffe, M.D. By a Reader of Biographies - 293
**The Doctor's Pulpit:** Hay-fever - 295
**The National Pension Fund** - 296
**Doctors in Fiction** - 297
**Notes and News.**—Hospital Appointments.—The Croydon Children's Convalescent Home.—The West Bromwich Self-supporting Dispensary.—Charity Cup Competitions.—Hospital Meetings and Reports.—Vacancies.—The Cork Dispensary.—The United Friendly Societies and Medical Charities.—The Jenny Lind Infirmary.—Concerts and Bazaars in Aid of the Hospitals.—Metropolitan Police Convalescent Seaside Home.—New Infirmary for Rutherglen.—The Bristol Royal Infirmary, etc., etc. - 298
**Annotations.**—Tweedledum or Tweedledee?—Wonderful Cure: Physic, Faith, or Neither?—Women and Work - 301
**The Editor's Letter-Box.**—Working Women: their Present and their Future.—Llanfairfechan Convalescent Home - 302
**Poetry.**—Alarming Result of Tight-lacing - 302
**A Friendly Atlas.** By W. J. E.—Chapters IV and V 303
**New Remedies:** Lanoline - 305
**Questions and Answers.**—Cottage Hospitals.—Home for a Lady - 305
**In- and Out-Patients' Hospital Diary** - 306
**The Children's Corner.**—Puzzles, Answers, etc. - vi
**Amusements with Profit** - vi

